# The dynamic resilience of urban labour networks

**DOI:** 10.1098/rsos.230214

**Published:** 2023-07-05

**Authors:** Xiangnan Feng, Alex Rutherford

**Affiliations:** Centre for Humans and Machines, Max Planck Institute for Human Development, Lentzeallee 94, Berlin 14195, Germany

**Keywords:** labour markets, skill spreading, complex networks, occupation influence

## Abstract

Both cities and markets are well understood as complex systems which are amenable to analysis using physically inspired methods. Cities have shown fascinating universality with size, while labour markets modelled as networks have considerable explanatory power. Labour markets are a particularly attractive domain of study in this context due to societal importance, the influx of high-resolution data as well as exogenous influence of automation. While much previous work has studied the economic characteristics of cities as a function of size and examined the exposure of urban economies to automation, this has often been from a static perspective. In this work, we examine the diffusive properties of labour markets and examine their variance across cities. More specifically, we identify the occupations which are most important in promoting the diffusion of beneficial or deleterious properties. To this end, we propose a new measure of node centrality empSI. We find that these properties of influence vary considerably with city size.

## 1. Introduction

Both cities and markets have a long history of study within complex systems. Cities have been described as agglomerative processes [1] and as universally scaling systems [2]. Labour markets, the allocation of workers with skills to tasks required to be performed by organizations, has led to the modelling of labour markets, skills and jobs as complex networks [3–9].

These two classes of system are, however, closely intertwined, suggesting a rich set of diffusive behaviours varying on urban substrates. This is attributable to locations advantageous for commerce, leading to economies of scale and increasing returns [10,11], and more recently encouraging the urban migration of workers to convene in centralized workplaces [12,13]. However, setting labour policies within urban levels (or indeed nationally or regionally) in an optimal way is both critical for prosperity yet challenging in the technical perspective [14].

With the development of complexity system, various network models have been applied on large datasets across various domains. Among these new trends, economies have been widely studied involving geographical features, skill demands, labour markets and economic profiles. Hosseinioun *et al.* analysed the skill portfolios to build the skill hierarchy network to study the latent structure behind the skill dependencies, diversification and specialization [15]. Neffke *et al.* built labour flow network to analyse the role of firms and entrepreneurs and their structure changes in different geographic and industrial divisions [16]. Hidalgo *et al.* used the trading data among countries to build the product space to study the patterns of products and adaption in different countries [17]. These frameworks are meaningful for various levels of economic agents: for policy-makers, finding out how to develop competitive products and services is one of their most crucial tasks; for firms, realizing their roles in local and global regions could help them extend their business both in geographical and industrial sectors; for employees, understanding the skill demands and industrial structure shifting will help make successful career plans and transitions.

Dynamic models and spreading patterns on networks have been a classic and important research topic in complexity science. Opinion dynamics, which represent the phenomenon of system dynamics created by local interaction among individuals, including group decision-making, marketing and spread of fake news, have received a lot of attention, with many concepts and models emerging [18]. With new datasets of human mobility collected from sensory devices and surveys [19], various models have been developed to track and predict the people’s movement and contagion events, including the spread of COVID-19 in recent years [20]. From the perspective of theory, to avoid the limits of classical networks, dynamical processes and percolation transitions on more complex systems like higher-order networks [21] and multiplex networks [22] have been studied to explore the phenomena like synchronization and consensus formation. These studies have pushed the boundary of research on network dynamics with many applications.

Although networks have been widely applied on economic datasets to analyse the labour markets, much previous work has focused on static properties of labour networks. However, labour markets are dynamic with flows of workers between jobs, of working practices between organizations and automation within firms and industrial sectors. Meanwhile, networks are natural representations for, not only how workers move between jobs that are mutually accessible based on skills, but also for diffusive processes by which technology spreads [23]. Little research work has attempted to apply the spreading models and theories on the economic data to study the labour market landscapes. Therefore, in this work, we focus on the resilience in an urban job network based on its network structure amenable to diffusion. This view is consistent with a large body of work examining the role of relatedness in the study of economic complexity [24]

We wish to make a strong link from our physically motivated modelling to implications that might be relevant for policy-makers. From this perspective, there are a number of attributes that might be expected to diffuse on a network of occupations. Some of these might help build an optimal environment, e.g. a gender-balanced workforce or safer and healthier working conditions, while some might be unfavourable, e.g. skill-based technological changes leading to the displacement of human labour. However, with knowledge of the degree to which a local job network will be able to promote or constrain diffusion and the nodes which are most influential in this network, policy-makers can more effectively drive the labour market to a desired state. For example, the adoption of company-wide policies protective of children and young people has been found to preferentially occur along network connections defined by supply chains [25].

**Figure 1.**
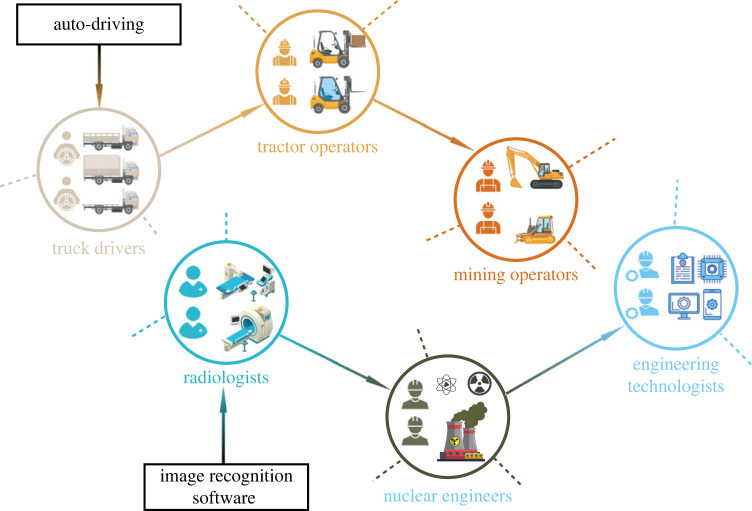
An illustration of workplace technologies spreading on an occupational network. Autonomous driving capability is first introduced for truck drivers, which subsequently spreads to impact tractor operators and then mining operators due to the similarities between these occupations. Likewise image recognition software introduced for radiologists spreads to nuclear engineers and engineering technologists. Not all occupations are drawn in this diagram; only those lying on one illustrative diffusive pathway.

In this manuscript, we focus on the mechanisms by which new technology diffuses (although this is amenable to the diffusion of other occupation-based norms). As shown in figure 1, when the workplace tasks of truck drivers are exposed to technologies such as autonomous-driving systems, this automation could spread preferentially to a related job, e.g. tractor operators. Subsequently other occupations similar to tractor operators with respect to skills could also be affected. However, this spreading phenomenon would not be limited to physical jobs. Image recognition software can assist radiologists diagnosing diseases in medical imagery, while the same technology could possibly be adopted by nuclear engineers to detect the operation of nuclear power plants; then engineering technologists could apply similar software onto other instruments to gain more powerful tools.

We wish to emphasize that the spread of technology might be beneficial or deleterious depending on the technology itself, as well as the stakeholders. A piece of technology might displace human labour completely leading to unemployment, but benefit a firm which is able to lower its wage bill. Likewise, a new piece of technology might well enable increased productivity in occupations depending on the efficiency of the technology in question [26].

Our contributions in this work are focused on providing a novel understanding of the structure and resilience of urban labour markets. More specifically, we (i) propose the employment-weighted spreading influence, a new measure of a node’s influence in a job network, (ii) we quantify the efficiency of diffusion based on a seeding strategy on this basis, (iii) we investigate how the cognitive and automatable nature of influential jobs changes with the size of the urban economy, and (iv) we uncover the most influential occupations, those that would optimize the efficacy of targeted policy interventions, across national urban centres.

## 2. Methods and data

### 2.1. Network construction

Firstly, occupation networks in each city are built. In the network, each node represents one occupation and each edge represents the similarity between the two occupations whose weight is given by skill similarity. We use the Jaccard similarity [27] to quantify the similarity between occupation *i* and *i*′,

2.1 $$\mathrm{SkillSimilarity}(i,i^{\prime })=\frac{\sum_{s}\min({\mathrm{onet}}_{i,s},{\mathrm{onet}}_{i^{\prime },s})}{\sum_{s}\max({\mathrm{onet}}_{i,s},{\mathrm{onet}}_{i^{\prime },s})},$$

where onet_*i*,*s*_ is the importance of skill *s* to occupation *i*. High skill similarity between jobs suggests workers make transitions more easily between them.

Since this similarity value between any occupation pair is not zero, a complete network will be built containing 774 nodes and 299 151 edges, which is the global network composed of all occupations in the USA. In each city, its occupation network is built in the same way with occupations whose employment is non-zero locally, which means each local occupation network is a complete sub-network of the global one.

### 2.2. Employment spreading influence

With a view of understanding the dynamic behaviours among various individuals on networks, a number of theories studying the interactions between nodes have been considered. These operate globally and locally to model the spreading processes, which usually focus on information [28] and epidemic spread [29]. A key issue is to find the ‘super-spreader’, namely the nodes with highest spreading abilities. Controlling these super-spreaders, that is to seed with them or isolate them purposefully, could lead to optimal spreading or immunization, respectively [30].

The spreading also happens on the labour markets and occupation networks. New technologies and skills from one occupation can potentially diffuse into other occupations, since new use cases beyond the original promote the introduction and adaptation of existing inventions. For example, the recording of speech was firstly invented by Edison to record the last words before people dying and books for blind persons, then it was adapted for office work. But even though Edison himself did argue against the use of this invention to record music, after around 20 years the recorders have totally revolutionized the whole music industry. If we view the skill similarity-based network from the perspective of spreading, the super-spreaders, namely the most influential occupations, would present the greatest ability to spread technologies or norms to the whole labour market. These occupations play crucial roles in leading and shaping the landscape of labour market. On one hand, if starting with them, new technologies improving efficiency could be adopted faster, which increases the productivity and economic growth. On the other hand, automation of skills and jobs could spread, leading to potentially negative effects such as short-term displacement of workers [31].

From this perspective, obtaining the most influential occupations could help regulate the whole labour market. For policy-makers, understanding which occupations are influential and making policies targeting these occupations and related skills could lead the labour market to more robust states with favourable economic outcomes.

Finding the optimal spreaders belongs to the non-deterministic polynomial (NP)-hard [32,33] problems generally. Interactions at different topological levels need to be studied [34,35] and multi-scale features tangling together increases the difficulty. It was studied that high-influence nodes are not always those whose degrees are highest [36]. By applying the cavity method [37] on this weighted network case [38], we define the spreading influence of occupation *i* in city *c*

2.2 $$S_{i}^{c}=\sum_{\;j\in N(i)}a_{ij}\sum_{k\in N(i),k\neq j}a_{ik}(d_{j}-1),$$

where *a*_*ij*_ = SkillSimilarity(*i*, *j*) is the edge weight between node *i* and *j*, *d*_*j*_ is the node degree. The summary and derivation with cavity method can be seen in appendix A.

Besides the network structure and skill similarity, the number of workers at each occupational node in the network is highly inhomogeneous, which has a strong influence on the labour market as a whole. Occupations with more employees should always be preferentially targeted by policies, since when new technologies are applied, they will have a large impact on the local or global labour market directly, either in a good way or in a bad way. Thus, when measuring the influence of occupations, we take into account the employment of occupation *i* in city *c*, denoted as ${\mathrm{emp}}_{i}^{c}$, to get the employment spreading influence

2.3 $$\begin{array}{rl} {\mathrm{empSI}}_{i}^{c} & =\log({\mathrm{emp}}_{i}^{c})S_{i}^{c}\\ & =\log({\mathrm{emp}}_{i}^{c})\sum_{\;j\in N(i)}a_{ij}\sum_{k\in N(i),k\neq j}a_{ik}(d_{j}-1). \end{array}$$

For each city, the labour market is described by its occupation network and the empSI of each occupation in that city is calculated. Occupations with high empSI suggest high influence on the local market in the perspective of spreading, which has a contribution due to both their structural spreading abilities or large employment numbers. Although the spreading models and the ‘super-spreader’ searching have been widely applied on various fields like epidemic and information spreading, it is the first time to consider and apply the percolation theory on the occupation networks. It is the first time to view the labour market in the respect of spreading to track and understand the dynamics of human resource and technologies. Comparing with other labour market research by statistical inference, this fresh perspective could help understand the complexity of labour market networks and other crucial ideas like regional relatedness [39]. Various stakeholders could benefit from these findings.

### 2.3. Data

In this research, we consider data from O*NET [40] and US Bureau of Labor Statistics [41]. Although similar structured data are increasingly available, e.g. in the EU [42], the use of O*NET is common in network studies of labour, and given the high quality of this data broken down by urban area, we proceed with this data. From this structured data, we build networks for each urban area as well as the whole nation. All the analyses are based on the built networks.

The O*NET data contain a set of standardized occupations and, for each occupation, a weighted importance against a set of standard skills. These data are compiled manually from expert interviews based on the skills that workers report. We consider the data from 2011 to 2020 as the standard occupational classification (SOC) system is consistent during this period. Thus the data we get includes 774 occupations globally and 120 items referred to as ‘skills’ including ability, knowledge and skill sections in the datasets are used to build networks. We use two-digit and six-digit SOC classifications for analysis.

The US Bureau of Labor Statistics provides employment data including occupations, employment numbers and salaries in each Metropolitan Statistical Area, commonly referred as a ‘city’. Around 380 cities are selected in the analysis. Not all occupations appear in every city, ranging from 93 to 733 occupations in each city. We build networks for each city based on the occupations found in it (as described in more detail below).

Other data include population data from US census [43] and occupation automation probability data from Frey & Osborne’s and Webb’s research [44,45]. Details about the automation data are introduced in appendix D. The occupations in different data sources are indexed by SOC codes.

## 3. Results

In this manuscript, we investigate the spreading processes of the labour market in each city. More specifically, we wish to identify the most influential occupations in each city network and determine how this relates to city size. For each city *c*, the ${\mathrm{empSI}}_{i}^{c}$ values of all occupations that are represented in the city workforce are calculated and those occupations with highest values are considered the most influential.

**Figure 2.**
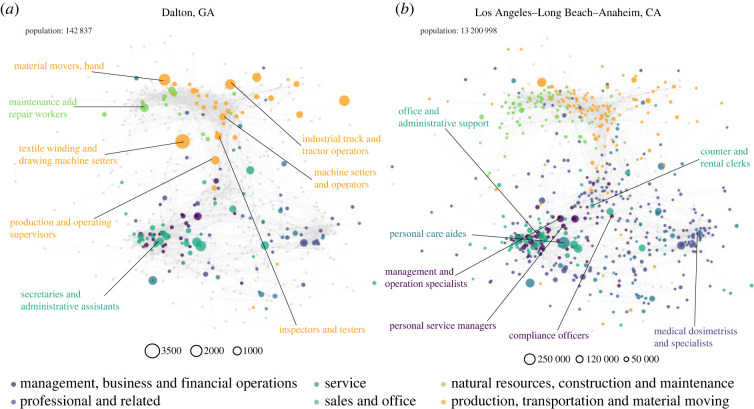
Two occupation networks of (*a*) Dalton, GA and (*b*) Los Angeles–Long Beach–Anaheim, CA. Nodes stand for occupations and edges stand for skill similarity between occupations. Node colours stand for sectors, and sizes indicate the employment numbers. The grey node indicates that this occupation does not appear in this city. Edges with weights higher than 0.7 are drawn, while the complete graph is applied in calculating. The labelled occupations are the different occupations in the two cities’ top-20 highest empSI occupations.

To begin, we compare the job networks from two cities with very different sizes: Dalton, GA (low population) and Los Angeles, CA (high population) in figure 2. The nodes (occupations) are linked by skill similarity (edges). We consider only the jobs that are found in each city, therefore the set of occupations (number of nodes) is typically different among cities. Each occupation has variable numbers of workers on that occupation that is represented by the size of the corresponding node. A selection of the most influential occupations are marked in each case. A distinction can be made between the two networks: in Dalton, the most influential occupations concentrate on the production and construction sectors, while in LA, the most influential ones belong to services, sales and clerical sectors.

**Figure 3.**
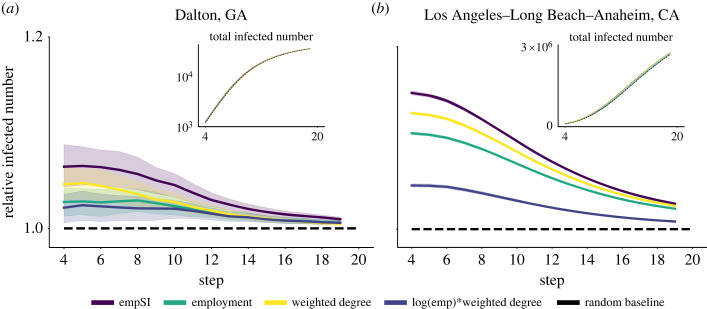
Simulation of a spreading process on the occupation networks of (*a*) Dalton, GA and (*b*) Los Angeles–Long Beach–Anaheim, CA. All results are the averaged values of 20 trials. In each simulation, 0.2% random agents of employment are selected by different strategies including highest empSI, highest employment numbers, highest weighted degrees and highest log(emp) × weighted degree as the starting seeds for spreading. Each node in the networks contains the same agent number as employment. At each step, each agent infects one of its neighbours with the probability given by their skill similarity. A strategy of random seed selection is applied as a baseline. The curves are the relative infected number and inside is the total infected number. The confidence intervals in Los Angeles are very small to distinguish, due to its large population.

To verify whether empSI meaningfully represents influential occupations, a simple spreading simulation within the susceptible-infected framework is conducted on the local networks of the two cities above (see model details in appendix B). Figure 3 presents the spreading processes starting with seed agents selected by different strategies. The random selection of the seeds is applied as a null model. As shown in the figure, seeding based on the empSI value leads to a faster spread than seeding at random or based only on employment or degree. These results show that with the proposed index, the most influential jobs could be found more accurately. These occupations work as the spreading hubs in the networks and could transit human sources and technologies faster to the whole labour market. They are functionally crucial for a healthy economic landscape and city development, which deserve more attention from various levels of stakeholders and policy-makers. As a verification, we made another spreading model analysis with susceptible-infectious-susceptible (SIS), which is given in appendix B.

### 3.1. Diversity and automation

Using the proposed empSI, a number of influential occupations in each city could be found, which usually are regarded as the most important and significant ones according to network centrality studies. In this section, based on the findings from proposed index, we investigate the relationship between the characteristics of an occupation: the cognitive nature and exposure to automation, and the occupation's importance within a city job network.

For each city, we compute the correlation between all occupations’ empSI values and cognitive scores, which is defined as the fraction of cognitive skills for each occupation [3], namely it is calculated by $\sum_{s\in \mathrm{cognitive}}{\mathrm{onet}}_{i,s}/\sum_{s}{\mathrm{onet}}_{i,s}$. In smaller cities, we find little correspondence between the two measures; in this case, the more influential occupations in small cities are just as likely to be cognitive or non-cognitive, whereas in larger cities, we find a reasonable correlation (*ρ* ≈ 0.3) between these measures. This is demonstrated by a clear positive trend between city size and the job-wise correlation in cognitive score and empSI ((*r*, *p*) = (0.32, <10^−9^)). Thus we expect that any occupational characteristic presented in more cognitive jobs, whether positive or negative, will spread more efficiently to other occupations in larger cities. Interestingly, in contrast to many economic indicators [10], we found no significant correspondence to a super- or sub-linear scaling with population.

**Figure 4.**
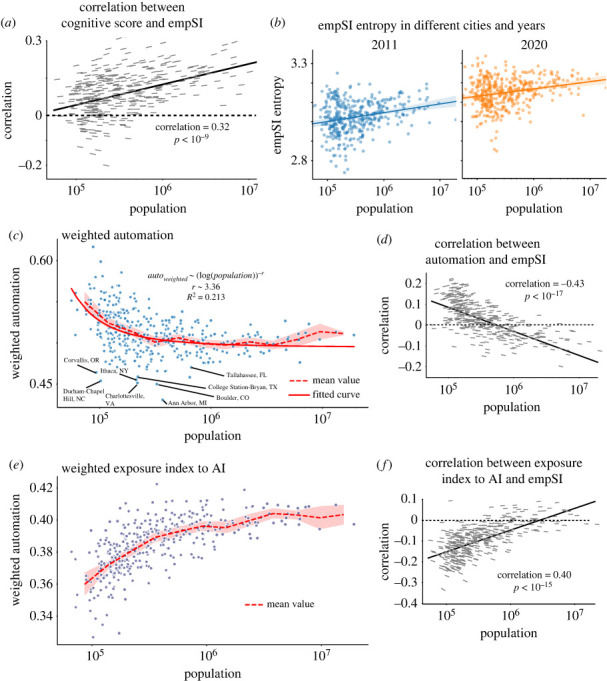
(*a*) Correlations between cognitive score values and empSI against populations in each city. The Pearson correlation coefficient is marked. Different slopes stand for different correlation values. (*b*) The empSI entropy of years 2011 and 2020 against populations. For each city, occupation empSI values are divided into same number of bins and their entropy is calculated. (*c*) Weighted automation probability by empSI against populations. The mean value is the average value in each bin, which is log-scale by populations. A log-power fitting is implemented on the data. On the left bottom, a group of college towns are labelled, featured by small-scale population and low-weighted automation probability. (*d*) Correlations between automation and empSI against population. (*e*) Weighted exposure index to AI from Webb’s research by empSI against populations. (*f*) Correlations between exposure to AI and empSI against population.

We note that this trend of increasing influence of cognitive jobs with city size also coincides with an increasing diversity of job influence, both with respect to city size and time. We calculate the Shannon entropy [46] of empSI in each city. Figure 4*b* demonstrates that the entropy of node influence across all occupations found in a city increases with population. Further, the level of diversity appears to be generally increasing between 2011 and 2020. Taken together, these results suggest that in larger cities, the correlation between the cognitive nature of jobs and their influence is not driven by a small number of highly influential cognitive jobs. Rather, the increasing uniformity in empSI, both over time and with city size, suggests that the choice of occupations for effective targeted policy interventions is not trivial (see a similar result related to spreading influence *S* and cognitive score in the appendix).

Next, we consider whether these trends in increasing diversity and more influential cognitive jobs in larger cities lead to increased *resilience* to automation specifically. In figure 4*c*, we evaluate the city-wise exposure to automation as measured by Frey & Osborne [44], weighted by empSI as below

3.1 $${\mathrm{auto}}_{\mathrm{weighted}}^{c}=\sum_{i}\frac{{\mathrm{empSI}}_{i}^{c}}{\sum \mathrm{empSI}}\times P_{\mathrm{auto}}(i),$$

where *P*_auto_(*i*) is the automation probability of occupation *i* from Frey & Osborne [44]. This value gives the probability of a job being susceptible to computerization, namely the risk of replacement from machines (see details in appendix D). The higher the value, the higher the replacement risk.

In common with previous findings [47], we see a decreasing exposure to automation with city size increasing when weighted by empSI: smaller cities face greater risks of automation. Meanwhile, at the left-bottom part of the figure, we observe a group of outliers, mostly corresponding to college towns including Ithaca, NY (Cornell University), Ann Arbor, MI (University of Michigan), Charlottesville, VA (University of Virginia) and Durham–Chapel Hill, NC (Duke University and North Carolina Central University). This phenomenon is consistent with the intuition that these college towns should be under low automation risk.

Further, the occupations that are most exposed to automation are less influential in larger cities. This result goes beyond a static picture of a city’s overall automation risk as measured by its present workforce. Rather, this index measures the degree to which an occupation is able to spread its own exposure to automation to other related occupations. Although this trend is relatively weak (from *ρ* ≈ 0.2 in the smallest cities to *ρ* ≈ −0.2 in the largest cities), it is consistent across all cities ((*ρ*, *p*) = (−0.425, <10^−17^)).

However, we find that this result is extremely sensitive to the exact measure of automation exposure. In figure 4*d*, we present the same weighted automation measure using data from [45], an index of exposure to AI specifically, which measure the overlap between job descriptions and pattern description related to AI. By contrast, we find the opposite trend in the relationship between the urban exposure to automation and population. Specifically, the weighted automation exposure has a positive correlation with city size ((*ρ*, *p*) = (0.40, <10^−17^)). Similar calculations are repeated based on other automation data [45,48], see appendix D.

The exposure of an occupation to automation is notoriously difficult to quantify, and the measures presented here all differ in what is measured. Here, we principally compare Frey & Osbourne’s [44] results as the most established measure on the one hand, and Webb’s [45] data as a more recent measure based on the similarity between occupation task descriptions and patent documents. Webb’s measure offers the key advantage of being validated on historical changes in workforce numbers, and is also broken down into three categories of AI, software and robotics.

Our results suggest that the cognitive jobs found preferentially in larger cities are more amenable to automation through the deployment of AI technology (as opposed to robots or software). AI technology can be more easily deployed in work environments where computers and data infrastructures are already common as well as through flexible cloud computing resources. The fact that no correspondence is found between city size and equivalent measures of automation exposure through robots and software support this.

In conclusion, results from our analysis suggest that in large cities, high-cognitive jobs tend to have higher influence on the labour markets. This conclusion agrees with the finding that larger cities tend to rely more strongly on high-cognitive skills with higher household income and annual wage [3], meanwhile larger cities always lead the development of economy and smaller cities follow the same pathway [49]. Also in larger cities, the labour markets present higher diversity, which is in agreement with the finding that there is less specialization in larger cities [50]. In respect to automation trend, urban labour market resilience has a nuanced relationship with city size and depends sensitively on the nature of the occupational automation risk. This relation is complicated since the influence of automation on labour market has been widely studied yet is still sophisticated [44]. Recent research suggests that in larger cities their higher job connectivity and diversity may bring higher resilience than in smaller ones [51]. We can conclude that larger cities are more exposed to AI-based automation technologies and that cognitive jobs will be able to diffuse occupational characteristics, possibly through targeted policy interventions, more efficiently. Still, AI-driven technologies are new trends [52] and history data may not help us capture and predict the future of labour markets accurately [53].

### 3.2. Landscape of US labour markets

Previous work has shown that the industry structure varies geographically [8,54,55]. For different cities, is there any pattern or particular combination of the influential occupations? What do the most common influential occupations and uncommon ones look like across cities?

**Figure 5.**
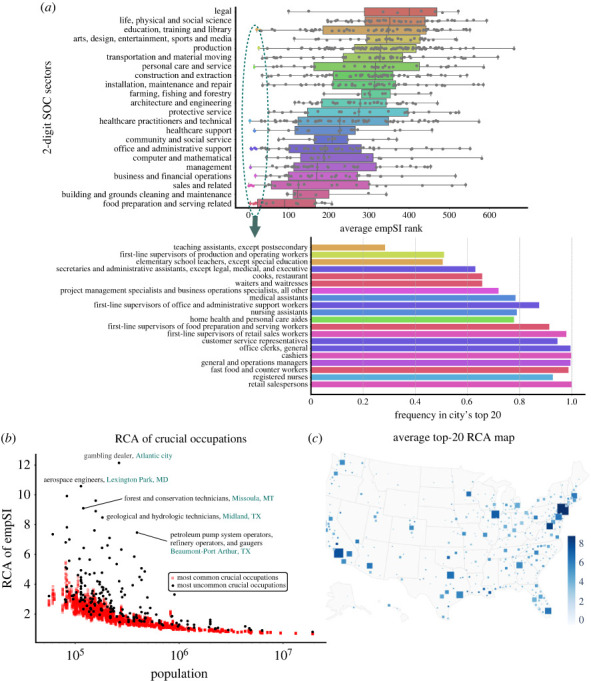
(*a*) Box plot of average empSI ranks of occupations in each sector. The two-digit SOC code is applied to divide occupations into different sectors, and different colours stand for different sectors. For each occupation, its empSI in every city is calculated and the average empSI rank is calculated. We select the 20 highest average rank occupations (marked by colourful nodes in the box plot) to count their frequencies of appearing in each city’s top-20 empSI. (*b*) Revealed comparative advantage (RCA) values of most common crucial occupations and uncommon crucial occupations against populations. The crucial occupations are defined as the top-20 empSI ones in each city. Several occupations are labelled. (*c*) The map of top-20 empSI RCA occupations in all cities. Square size stands for populations and colour stands for the value.

To find the common pattern of influential occupations, in each city, we sort all active occupations by their local empSI and for each occupation its average rank is calculated across the whole nation. We investigate the occupations with highest average empSI ranks by counting their frequency of ranking top 20 in local labour markets. As shown in figure 5*a*, we find that occupations in different sectors demonstrate different rank patterns. There are some occupations ranking among the highest in almost all the cities, suggesting that they are crucial and influential everywhere, which concentrate in sales, services, health and office sectors, like retail salespersons, registered nurses, cashiers and office clerks. Most of these occupations are regarded as basic life-supporting and economic-activity ones. Although some of them do not have a high essential score [56,57] and their automation risks vary [44,45,48], these occupations, due to both large employment numbers or high spreading abilities based on skill similarities, play influential roles across all cities. For policy-makers, these occupations are crucial since they are fundamental and prevailing for local economy, and global policies targeting these occupations may cause similar effects around the whole country.

While there are a group of common influential occupations across the whole nation, one might wonder what makes the labour market particular to the cities? Previous work has shown that features vary with the scale of cities, including wages, patent activity and characteristic industries [49,58,59]. Are the existing influential occupations specific to particular cities? To verify this, we calculate the revealed comparative advantage (RCA) [24] of occupation empSI in each city

3.2 $${\mathrm{RCA}}_{i}^{c}=\frac{{\mathrm{empSI}}_{i}^{c}/\sum_{i}{\mathrm{empSI}}_{i}^{c}}{\sum_{c}{\mathrm{empSI}}_{i}^{c}/\sum_{i}\sum_{c}{\mathrm{empSI}}_{i}^{c}}.$$

This index could help find the relative advantage of an occupation, namely occupations with high ${\mathrm{RCA}}_{i}^{c}$ will present high influence on local labour market and this influence is identical to this city—in cities other than *c*, these occupations are either playing negligible roles or not active.

We check the occupation frequencies in every city and obtain the most common and uncommon crucial occupations. In figure 5*b*, we present their ${\mathrm{RCA}}_{i}^{c}$ values. It could be observed that some occupations, featured by high ${\mathrm{RCA}}_{i}^{c}$, are influential in only one or several cities, like gambling dealer in Atlantic City which is famous for gambling industry, aerospace engineers in Lexington Park in Maryland, where the NASA Goddard Space Flight Center is close, and geological and petroleum-related work in Midland and Port Arthur in Texas, where the oil sources were discovered. These high ${\mathrm{RCA}}_{i}^{c}$ occupations present strong geographical identities (see in figure 5*c*). Similar identities of which could be found in the map of job embeddedness [51], distribution of general skill scores [15], and expected job impact from automation in every city [47]. These geographically identical occupations deserve more attention from policy-makers, since they could play special roles across the whole nation, e.g. output special productions and deal with particular issues.

Our findings, in respect to higher diversity in larger cities, agree with conclusions from recent literature. Bettencourt *et al*. claimed that the coexistence of greater individual specialization and increases in overall diversity is the optimal [50]. In our findings, both larger and smaller cities present specialization abilities in different ways: while in smaller cities geographical specialization features are performed by some specific high ${\mathrm{RCA}}_{i}^{c}$ value occupations, in large cities like New York and Los Angeles, high average ${\mathrm{RCA}}_{i}^{c}$ values of top influential occupations suggest there are also rare but crucial occupations. Combining the high exposure to AI in larger cities in our previous results, the AI may be the next great opportunity for them to keep the increase of diversity and economic vigour. Nevertheless, elucidating the intrinsic mechanisms and dynamics underneath the labour market diversity and specialization still requires further research. Some researcher found that the job connectivity is crucial for diversity and resilience [51], while network indices like nestedness is applied to study the dynamics of industrial ecosystem [60]. More research with all-around models and detailed analysis on new data could be expected in the future.

In summary, it has been shown from this empSI perspective that there are some occupations that appear and play crucial roles in every city, which have high average empSI ranks and usually focus on life and health sectors, like sales, services, health and office. They are supporting the everyday routine life in the global nation and prevailing in many professions. At the same time, there are some unique occupations in some cities, which are deeply related to the local economy structures and city geographical features like college towns and nature resource centres, being crucial and influential locally. They tend to have high empSI RCA values and deserve specialized attention and policies. These two aspects of crucial occupations, the globally crucial ones and locally crucial ones, make up the backbones of US labour market landscape.

## 4. Conclusion and discussion

The adoption of workplace behaviours, like many other social norms, can have positive and negative effects. In the context of workplace norms, the adoption of safer working conditions would be beneficial for workers whereas the adoption of new technology could potentially be a negative development for both workers and policy-makers. Given the many successes of describing complex social systems, such as labour markets, as complex networks, it is natural to consider the diffusive properties of labour markets. This provides an attractive opportunity for policy-makers to take advantage of this diffusive property to encourage (inhibit) the faster (slower) adoption of positive (negative) attributes on the network of occupations.

From the perspective of spreading, we have proposed an employment spreading index to measure the occupation influence on occupation networks. For each city, an occupation network is built based on skill similarities between jobs and the most influential occupations are obtained by the index. We investigate the systematic effect of city size on the susceptibility of jobs. We find that cognitive jobs are consistently more influential as city population increases. Regarding the effect of automation specifically, we find that the relationship with occupational influence is more nuanced. The Frey & Osbourne [44] measure of exposure to automation suggests that larger cities will be more resilient to automation as the influential occupations are those with a lower exposure. Conversely, Webb’s [45] measure of exposure to AI specifically suggests the opposite trend; larger cities have their more influential occupations more exposed.

Considering influential occupations on a global level, by aggregating the most influential occupations across all cities, we are able to rank occupations. We find that the top jobs tend to include an element of physical and socio-cognitive work e.g. nurses, cashiers and customer service representatives. These results suggest that these mixed-nature jobs should be given special attention by policy-makers when considering how to manage labour market change, whether technological in nature or not.

In this work, we have considered the occupation networks linked by the similarity of the tasks required. However, behaviours such as the adoption of automation technologies are able to diffuse on many network substrates; between the successive jobs of individual workers, between firms in common sectors in physical space or along supply chains. Considerable explanatory power has been found in novel combinations of these, e.g. geo-industrial networks [8] and supply chain networks [61]. It is likely that the true dynamics involve some combination of these into a multiplex network [62], on which the cooperative spreading of more than one social norm would diffuse [63,64]. More related studies of the diffusion mechanism on labour market networks could bring deeper understanding on them. A key challenge will be how to apply new strategies to capture these patterns, like research on high-order interactions [65,66] and structures including motif [67,68] and graphlet [69]. The occupation network structure deserves more explorations in the future.

Our study is constrained by the availability of public data. More research into the spreading processes and models deserve further attention. If the flows among occupations of specific technology or human resource could be accurately tracked, the trends of automation and skill demands would be studied much more clearly, which would tremendously help depict the landscape of labour market. This study relies on much more detailed data. Higher fidelity data would also allow for more careful validation of these findings. In our research, we make conceptual simplifications that the occupational network is static for the timescale for diffusion to take place. Implicitly, this means that we ignore the fact that the labour market is dynamic: new occupations are generated every year and for many occupations new skills and working contents are introduced [70,71].

## Figures and Tables

**Figure 6.**
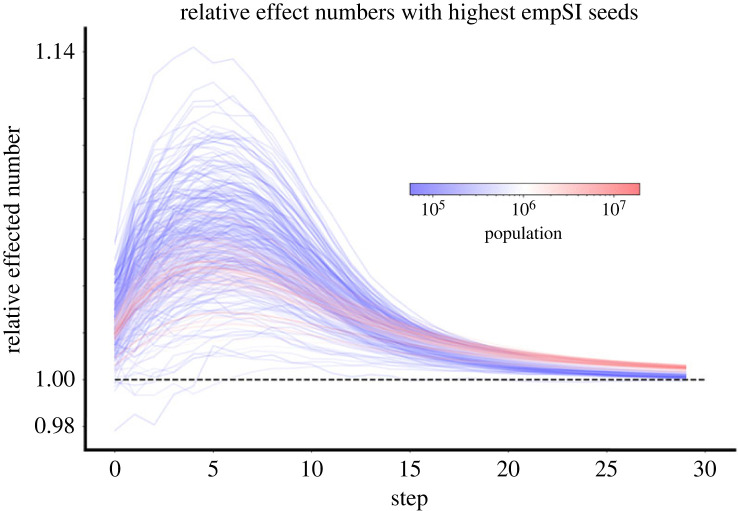
Spreading simulation with high empSI seeds against random results. Cities with different populations are marked by different colours, with blue standing for small cities and red large cities. Each curve is the average results of 20 simulation trials.

**Figure 7.**
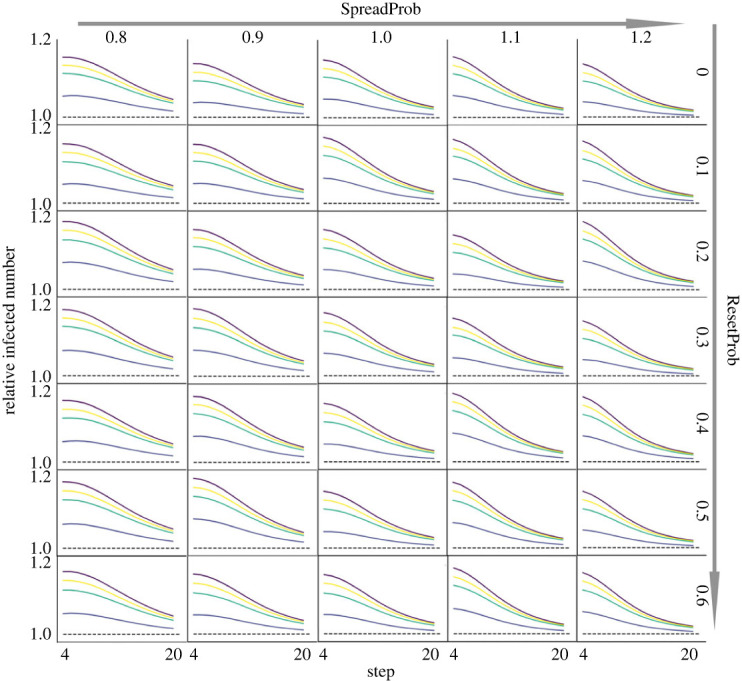
Simulation of SIS process on the occupation networks of Los Angeles–Long Beach–Anaheim, CA. All results are the averaged values of 20 trials. In each simulation, 0.2% random agents of employment are selected by different strategies including highest empSI, highest employment numbers, highest weighted degrees and highest log(emp) × weighted degree as the starting seeds for spreading. Each node in the networks contains the same agent number as employment. At each step, each agent infects one of its neighbours with the probability given by their skill similarity. A strategy of random seed selection is applied as a baseline. The curves are the relative infected number and inside is the total infected number. The confidence intervals in Los Angeles are very small to distinguish, due to its large population. Cases with different values of *ResetProb* and *SpreadProb* mentioned in algorithm 2 are presented in the simulations.

**Figure 8.**
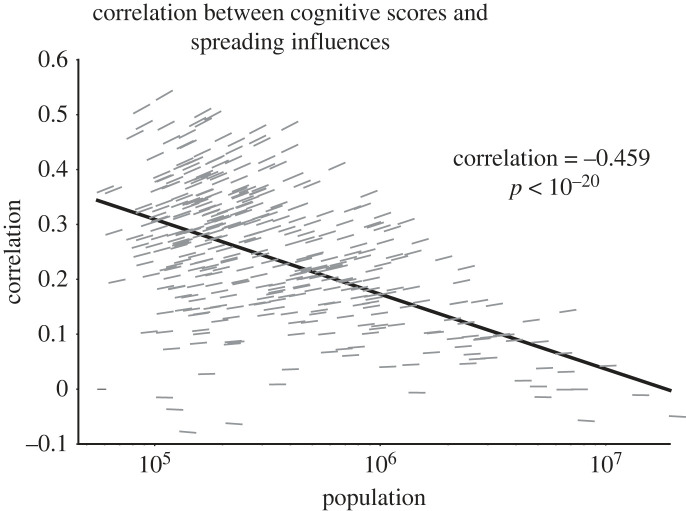
Correlations between cognitive score values and spreading influence *S* against populations in each city. The Pearson correlation coefficient is marked. Different slopes stand for different correlation values.

**Figure 9.**
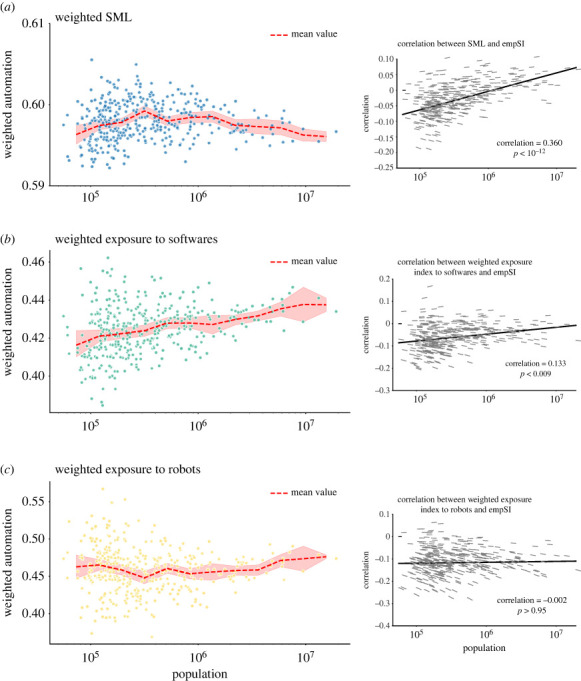
(*a*) Weighted suitability for machine learning (SML) from Brynjolfsson and Mitchell’s [52] research work with empSI. The correlation between SML and empSI is drawn on the right side. (*b*) Weighted exposure score to software patents. (*c*) Weighted exposure score to robot patents.

## Data Availability

In this manuscript, only public data are used. The data could be used under an open access license. All the analysis, data and code could be downloaded with this link: https://github.com/Roland-Feng/empsi; the Zenodo link for the manuscript is https://doi.org/10.5281/zenodo.8026260 [72].

## Appendix A. Derivation of spreading influence

This result by 1RSB is assumed on sparse network [37,38], especially on locally tree-like networks where there are not many loops, yet it still could work on more dense networks. In our research, we applied the weighted networks to model the occupation networks and the conclusion still works. In many studies related to cavity method and message passing, it is common to extend the conclusions from locally tree-like networks to more complicated ones [73–75]. Usually, it is too complicated to do the derivations on networks with many loops, while results from tree-like structure networks provide a rigorous bound to results from networks containing loops [76]. Recently, there are some studies attempting to remedy and rectify this problem [77,78], which deserve further exploration in future research.

By using message passing formula, the dynamical systems of spreading patterns on networks could be built. To guarantee a stable solution of this system, the largest eigenvalue of the corresponding linear operator which is represented using non-backtracking matrix [79] with directed links should be less than one. In this way identifying super-spreaders problem is transferred into solving the stable solution problems. To estimate the largest eigenvalue, the power method from numerical analysis [80] is applied to make calculations. After derivation, the largest eigenvalue could be estimated by each node contribution to it, which is regarded as the influence of node on the network. The larger its contribution, the greater its influence in spreading.

Under the sparse network assumption, for a network *G* with node set *V* and edge set *E*, its adjacency matrix *A* is defined as

A 1 $$A_{ij}=\left\{ \begin{array}{cc} 1 & \mathrm{if}\;v_{j}\;\mathrm{and}\;\mathrm{are}\;\mathrm{connected}\\ 0 & \mathrm{otherwise}. \end{array}\right.$$

In a weighted network case, we use *a*_*ij*_ to stand for the weight of edge between node *v*_*i*_ and *v*_*j*_.

The maximum spreading problem equals the optimal percolation problem [36], which is how to break down the major component in networks with minimal set of nodes removed. Let *c*_*i*→*j*_ denote the probability of node *v*_*i*_ belonging to the major component with node *v*_*j*_ removed. For a tree-like weighted network, this relation could be formulated as

A 2 $$c_{i\to j}=n_{i}[1-\prod_{h\in \partial i,h\neq j}(1-c_{h\to i}a_{ih}A_{ih})],$$

where *n*_*i*_ = 0 stands for node *v*_*i*_ being an optimal spreader and not if *n*_*i*_ = 1 and *h* ∈ ∂*i* means the neighbours of node *v*_*i*_.

Based on knowledge from dynamic system, the above system will have a stable solution if the largest eigenvalue of linear operation R is smaller than 1 [81]. Here, the R is a 2|*E*| × 2|*E*| matrix and each row and column corresponds to a one-direction edge. The matrix R is defined as

A 3 $$R_{i\to j,k\to l}={\frac{\partial c_{i\to j}}{\partial c_{k\to l}}|}_{c_{i\to j=0}},$$

where *i* → *j* and *k* → *l* stand for the edges between *v*_*i*_ and *v*_*j*_ and *v*_*k*_ and *v*_*l*_ with directions. R could be calculated by non-backtracking matrix M [79]

A 4 $$R_{i\to j,k\to l}=n_{k}a_{ij}M_{i\to j,k\to l},$$

where

A 5 $$M_{i\to j,k\to l}=\left\{ \begin{array}{cc} 1 & \mathrm{if}\;i=j,k\neq l\\ 0 & \mathrm{otherwise}. \end{array}\right.$$

Thus, we have

A 6 $$R_{i\to j,k\to l}=n_{k}a_{ij}A_{ij}A_{kl}\delta_{\;jk}(1-\delta_{il}),$$

where

A 7 $$\delta_{ij}=\left\{ \begin{array}{cc} 1 & \mathrm{if}\;i=j\\ 0 & \mathrm{otherwise}. \end{array}\right.$$

In the equation above, *A*_*ij*_, *A*_*kl*_ and *δ*_*jk*_ suggest that there is a path *v*_*i*_ → *v*_*j*_ → *v*_*k*_ → *v*_*l*_, and *δ*_*il*_ makes sure that it is a non-backtracking path.

Let *λ*(**n**) be the largest eigenvalue of R where **n** = (*n*_1_, *n*_2_, …, *n*_|*V*|_) indicates which nodes are selected as optimal spreader. We apply the power method [80] from numerical analysis to approximate *λ*(**n**). Let **w**_0_ be a vector satisfying that it has non-zero projection on the direction of *λ*(**n**)’s eigenvector and let **w**_*r*_(**n**) be

A 8 $$w_{r}(n)=R^{r}w_{0}.$$

Then, according to power method, we could get

A 9 $$\lambda (n)=\lim_{r\to \infty }\lambda_{r}(n)=\lim_{r\to \infty }{\left(\frac{\left|w_{r}(n)\right|}{\left|w_{0}\right|}\right)}^{1/r},$$

where

A 10 $$|w_{r}(n){|}^{2}=\langle w_{r}(n)|w_{r}(n)\rangle =\langle w_{0}|{\left(R^{r}\right)}^{T}R^{r}|w_{0}\rangle .$$

When *r* = 1, the approximated eigenvector **w**_1_ is

A 11 $$|w_{1}(n)\rangle =R|w_{0}\rangle .$$

Let |**w**_0_〉 = |**1**〉, the left vector could be calculated as

A 12 $$\begin{array}{rl} {\;}_{i\to j}\langle w_{1}(n)| & =\sum_{k\to l}{\;}_{k\to l}\langle w_{0}|R_{k\to l,i\to j}\\ & =\sum_{k\to l}n_{i}a_{kl}A_{kl}A_{ij}\delta_{li}(1-\delta_{kj})\\ & =n_{i}A_{ij}\sum_{k\to l}a_{kl}A_{kl}\delta_{li}(1-\delta_{kj}), \end{array}$$

and the right vector

A 13 $$\begin{array}{rl} {\left|w_{1}(n)\right\rangle }_{i\to j} & =\sum_{k\to l}R_{i\to j,k\to l}{\left|w_{0}\right\rangle }_{k\to l}\\ & =\sum_{k\to l}n_{j}a_{ij}A_{ij}A_{kl}\delta_{\;jk}(1-\delta_{il})\\ & =n_{j}a_{ij}A_{ij}\sum_{k\to l}A_{kl}\delta_{\;jk}(1-\delta_{il}). \end{array}$$

So the norm of **w**_1_(**n**) is

$$\begin{array}{rl} |w_{1}(n){|}^{2} & =\sum_{i\to j}{\;}_{i\to j}\langle w_{1}(n){\left|w_{1}(n)\right\rangle }_{i\to j}\\ & =\sum_{i\to j}n_{i}n_{j}a_{ij}A_{ij}\times \sum_{k\to l}a_{kl}A_{kl}\delta_{li}(1-\delta_{kj})\sum_{k\to l}A_{kl}\delta_{li}(1-\delta_{kj}). \end{array}$$

Let $M_{i\to j}=\sum_{k\to l}a_{kl}A_{kl}\delta_{li}(1-\delta_{kj})$ and $N_{i\to j}=\sum_{k\to l}A_{kl}\delta_{\;jk}(1-\delta_{il})$, then

A 14 $$|w_{1}(n){|}^{2}=\sum_{i\to j}n_{i}n_{j}a_{ij}A_{ij}M_{i\to j}N_{i\to j},$$

and the approximation to largest eigenvalue is

A 15 $$\lambda_{1}(n)={\left(\frac{1}{2|E|}\sum_{i\to j}n_{i}n_{j}a_{ij}A_{ij}M_{i\to j}N_{i\to j}\right)}^{1/2}.$$

For *r* = 2, the left vector _*i*→*j*_〈**w**_2_(**n**)| and right vector |**w**_2_(**n**)〉_*i*→*x*=*j*_ could also be calculated

$$\begin{array}{rl} {\;}_{i\to j}\langle w_{2}(n)| & =\sum_{k\to l}{\;}_{k\to l}\langle w_{1}|R_{k\to l,i\to j}\\ & =\sum_{k\to l}n_{i}n_{k}a_{kl}A_{kl}A_{ij}\delta_{li}(1-\delta_{kj})M_{k\to l}\\ & =n_{i}A_{ij}\sum_{k\to l}n_{k}a_{kl}A_{kl}\delta_{li}(1-\delta_{kj})M_{k\to l}; \end{array}$$

and

$$\begin{array}{rl} {\left|w_{2}(n)\right\rangle }_{i\to j} & =\sum_{k\to l}R_{i\to j,k\to l}{\left|w_{1}\right\rangle }_{k\to l}\\ & =\sum_{k\to l}n_{l}a_{kl}A_{kl}N_{k\to l}n_{k}a_{ij}A_{ij}\delta_{\;jk}(1-\delta_{li})\\ & =a_{ij}A_{ij}\sum_{k\to l}n_{k}n_{l}a_{kl}A_{kl}\delta_{\;jk}(1-\delta_{li})N_{k\to l}. \end{array}$$

Thus, the second-order eigenvector is

$$\begin{array}{rl} |w_{2}(n){|}^{2} & =\sum_{i\to j}{\;}_{i\to j}\langle w_{1}(n){\left|w_{1}(n)\right\rangle }_{i\to j}\\ & =\sum_{i\to j,k\to l}n_{i}n_{j}n_{k}n_{l}a_{ij}a_{ki}a_{lj}A_{ij}A_{ki}A_{lj}M_{k\to i}N_{\;j\to l}, \end{array}$$

and the approximated eigenvalue is

A 16 $$\lambda_{2}(n)={\left(\frac{1}{2|E|}\sum_{i\to j,k\to l}n_{i}n_{j}n_{k}n_{l}a_{ij}a_{ki}a_{lj}\times A_{ij}A_{ki}A_{lj}M_{k\to i}N_{\;j\to l}\right)}^{1/4}.$$

A more generalized form of |**w**_*r*_| and *λ*_*r*_(**n**) could be got

$$\begin{array}{rl} \left|w_{r}(n)\right| & =\sum_{i_{1}\to j_{1},i_{2}\to j_{2},\ldots ,i_{r}\to j_{r}}a_{i_{1}j_{1}}M_{i_{2}\to i_{1}}N_{\;j_{r-1}\to j_{r}}\\ & \times \prod_{k=1}^{r}n_{i_{r}}n_{\;j_{r}}\prod_{k=1}^{r-1}a_{i_{k}i_{k+1}}a_{\;j_{k}j_{k+1}}A_{i_{k}i_{k+1}}A_{\;j_{k}j_{k+1}} \end{array}$$

and

$$\begin{array}{rl} \lambda_{r}(n) & =(\frac{1}{2|E|}\sum_{i_{1}\to j_{1},i_{2}\to j_{2},\ldots ,i_{r}\to j_{r}}a_{i_{1}j_{1}}M_{i_{2}\to i_{1}}N_{\;j_{r-1}\to j_{r}}\\ & {\times \prod_{k=1}^{r}n_{i_{r}}n_{\;j_{r}}\prod_{k=1}^{r-1}a_{i_{k}i_{k+1}}a_{\;j_{k}j_{k+1}}A_{i_{k}i_{k+1}}A_{\;j_{k}j_{k+1}})}^{1/2r}. \end{array}$$

In our research, we use the *r* = 1 case. The spreading influence of node *v*_*i*_ is regarded as the contribution of all value of edge between *v*_*i*_ and *v*_*j*_ contained in *λ*_1_(**n**), namely

A 17 $$\begin{array}{rl} S_{i} & =\sum_{\;j\in N(i)}n_{i}n_{j}a_{ij}A_{ij}M_{i\to j}N_{i\to j}\\ & =\sum_{\;j\in N(i)}a_{ij}\sum_{k\in N(i),k\neq j}a_{ik}(d_{j}-1). \end{array}$$

## Appendix B. Spreading simulation

To examine the validation of employment spreading influence, we apply an agent-based spreading simulation. We want to verify if the infection starts with high empSI occupations will it spread through the whole network faster.

The complete occupation networks in Dalton, GA and Los Angeles, CA are used to simulate the infection process. In the networks, nodes stand for occupations and edges are weighted by skill similarity. In each node, there are agents with the same number to employment of corresponding occupation.

We select 0.2% agents in Los Angeles and Dalton network scattered uniformly into top-20 occupations as initial infected agents. If the employment of one selected job is lower than the infection number, we will take the employment number as the infected one, corresponding to the case of occupations with high similar to others but low employment numbers. For each step, each infected agent (source agent) selected one agent from its neighbour occupations, which we call ‘target agent’. If the target agent is not infected, the source agent infects the target with the probability of skill similarity; if the target agent is already infected, the source agent will do nothing. After each step, all the infected agents will become source agents and could start to infect others.

In different simulations, the initial infected agents are selected by highest empSI, highest employment and highest weighted degree. At the same time, a simulation with random selected initial infected agents is implemented to work as the baseline. For each simulation, 20 trials are implemented and average results are calculated. We present the relative infected numbers in each step against the baseline in the main paper.

The simulation process is described in algorithm 1.

Results of spreading simulation with highest empSI jobs as seeds against random results in all cities are presented in figure 6. A subtle negative correlation between spreading speed and city scale could be observed: the spreading starting with high empSI seeds tends to be slower than in smaller cities. We infer that this is due to the higher complexity and more diverse occupation structure in larger cities. Further exploration in the spreading dynamics geo-variation could be expected in the future.

To examine the spreading patterns in the case of more complex contagions, we conducted a similar spreading processes with SIS model, namely during the spreading progress, the agent will not transfer the skill to others with a certain possibility at next step. We introduced a parameter *ResetProb* to stand for the probability that agent will ‘recover’ temporarily from the skill and not infect others. Meanwhile, we introduced another parameter *SpreadProb* to tune the spreading probability. The specific process is described in algorithm 2.

For various values of *ResetProb* and *SpreadProb*, we conducted the spreading simulation on LA occupation network (see figure 7). We find that although the derivation of empSI is based on SI model, this index still performs well in SIS cases. In real world, the spreading process could be much more complex, which deserves further research to build models with more precise details and dynamical patterns.

## Appendix C. Correlation between cognitive scores and spreading influences

In the main text, we presented relationships between empSI and cognitives in different cities. Here, a similar work is done for spreading influence *S* and cognitive scores (see figure 8). We find that there is a clear negative trend between the job-wise correlation in cognitive score and *S*. This is despite smaller cities generally having fewer workers in cognitive jobs, suggesting that this is driven by the more numerous *non-cognitive* jobs being relatively *un-influential*.

## Appendix D. Weighted automation

Automation is a widely studied topic in labour market research. It measures the risk of jobs being substituted by machine, which is a crucial issue especially in this AI era. In the main text, we calculate the weighted automation by empSI with occupation data from Frey and Osborne’s research work [44]. In their work, automation possibility of 70 occupations are selected to be hand-labelled by a group of machine learning researchers as 0 for impossible to be replaced by machine and 1 for will be replaced by machine, which are used as the labels in training set. Then nine variables-related computerization bottleneck from O*NET [40] describing the occupations are selected as features to be trained by a Gaussian process classifier. In this way, the automation and computerization probability of other occupations from O*NET could be estimated by the trained classifier.

There are some other research works about automation probability estimation. Brynjolfsson *et al.* designed a rubric with 23 distinct statements to evaluate the ‘suitability for machine learning’ (SML) of 2069 direct work activities which the occupations could be mapped to, to estimate the SML scores of occupations [48]. Webb calculated the overlaps between job task description texts and patent texts to measure the exposure of occupations to automation, in the perspectives of software, industrial robots and artificial intelligence. These indices are measures of the dependence of jobs to corresponding technologies. They both got meaningful data and conclusions [45].

Based on the automation data from this research, we also calculate their corresponding weighted automation by empSI, and results are presented in figure 9. A similar conclusion could be seen from the SML data and larger cities perform higher resilience against automation. The curves from Webb’s data look opposite and larger cities present higher weighted values.

## Appendix E. Notes on RCA values

When we calculate the RCA values of empSI, there are some occupations with very high RCA values, mostly in the natural sources and production sectors. These high RCA values are mostly because of insufficient data, since these occupations do appear in other cities yet their employment numbers are not available. It is hard to decide whether the RCA values follow some certain distributions (like power law [82], which will make the average meaningless). So in our research, we remove some extreme high RCA values.
